# Tracing the Fate of the Northern Bald Ibis over Five Millennia: An Interdisciplinary Approach to the Extinction and Recovery of an Iconic Bird Species

**DOI:** 10.3390/ani12121569

**Published:** 2022-06-17

**Authors:** Johannes Fritz, Jiří Janák

**Affiliations:** 1 Waldrappteam Conservation & Research, 6162 Mutters, Austria; 2 Department of Behavioral and Cognitive Biology, University of Vienna, 1090 Vienna, Austria; 3 Czech Institute of Egyptology, Charles University, 11000 Prague, Czech Republic; jiri.janak@ff.cuni.cz

**Keywords:** Northern Bald Ibis, climate change, restoration, translocation, migration, interdisciplinary approach, ancient Egypt

## Abstract

**Simple Summary:**

Once widespread around the Mediterranean region, the Northern Bald Ibis (*Geronticus eremita*) became one of the rarest birds in the world. In this paper, we trace the history of the species through different epochs up to the present. A particular focus is placed both on its life in and disappearance from ancient Egypt, where the bird attained significant cultural importance, and on the modern endeavour to re-wild and restore the species. The Northern Bald Ibis is an outstanding example of how an interdisciplinary cultural-historical and natural-scientific approach significantly promotes the interpretation of historical evidence as well as helps implement current rewilding and restoration efforts.

**Abstract:**

We trace the history of the endangered Northern Bald Ibis through different epochs to the present. A particular focus is placed on its life in and disappearance from ancient Egypt, where the bird attained great cultural and religious significance, and on the modern endeavour to re-wild the species. Due to the characteristic appearance, behaviour and habitat of the species as well as its need for open foraging areas, a close mutualistic relationship between humans and the birds was formed in ancient Egypt, as in other cultures. A clear benefit for the Northern Bald Ibis was the availability of feeding habitats, which were cleared by humans for farming or grazing. The benefit to people was rather cultural because the bird attracted religious veneration or symbolic meanings from ancient Egypt to medieval Europe. The proximity to humans, however, carried a high risk as well. We discuss various types of impact (including human impacts as well as climate change) as triggers for the extinction of the species. The evidence for a triple disappearance of the Northern Bald Ibis (around 2000 BCE, around 1600 CE and in modern time) represents a unique basis for studying both the bird’s habitat preferences and its vulnerability. This is because different, mainly anthropogenic, causes stood behind these three historical disappearances, although the disappearances in all three epochs occurred during a period of climate change.

## 1. Introduction

There is a growing consensus that the rapidly increasing challenges of biodiversity conservation and restoration cannot be overcome without a thorough knowledge of human society and its history [1]. In an interdisciplinary approach, both the humanities and natural sciences can promote the sustainability and effectiveness of conservation measures if certain obstacles are overcome [2]. At the same time, successful cooperation between the two scientific fields can provide a much needed broader picture and new interpretations of historical events and developments [3,4].

The story of the Northern Bald Ibis (*Geronticus eremita*) combines current conservation needs and cultural-historical research questions in an exemplary way. This species is one of the most threatened birds in the world; it was listed as “critically endangered” in the IUCN Red List for 24 years, before being downgraded to “endangered” in 2018 due to extensive protection efforts [5]. Its unique appearance, conspicuous behaviour and diverse flight characteristics make this migratory bird a noticeable creature both on the ground and in the air. Moreover, humans created preferable habitat for the species by cultivating fields and meadows in different regions and epochs; this inevitably made the history and fate of this species closely interwoven with that of the human cultures with which it interacted and shared habitats [6,7,8].

The oldest and most extensive historical evidence on the Northern Bald Ibis and its sudden disappearance comes from ancient Egypt, where its depiction was used in Egyptian scripts as a hieroglyph with the easily recognizable characteristic shape and other features of the bird’s body [4]. As a natural model for the hieroglyphic sign for “blessed ancestor spirits” (Akh), the bird attained great religious and symbolic significance (for more information, see the Appendix A).

In this paper, we trace the history of the Northern Bald Ibis over five millennia through different periods from ancient Egypt to the present. Current knowledge of Egyptology is combined with biological and ecological research and current protection measure. Ancient evidence witnesses a decline caused by higher human activity and responses to a climate change, on the other hand late medieval and modern evidence show other reasons of the decline. The paper combines all available data (both ancient, later, and modern) to shed light on ecological and cultural-historical peculiarities of the species and, mainly, on the reasons for the decline of the bird’s population in different regions. This interdisciplinary and comparative approach helps generate strategies for current and planned protection measures for the Northern Bald Ibis and other endangered migratory bird species.

## 2. Materials and Methods

### 2.1. Biology and Ecology

This paper specifically refers to studies on Northern Bald Ibis behaviour and ecology done within the framework of a European LIFE reintroduction project (LIFE+12-BIO_AT_000143; www.waldrapp.eu, accessed on 22 June 2014) and the underlying feasibility study [5]. These projects aim to establish a migratory population in Central Europe with a migration tradition to southern Tuscany. A steadily increasing number of wild-living birds (199 individuals in late 2021) migrate between the common wintering site and different breeding sites north and south of the Alps [9].

In the 20 years since the beginning of the reintroduction of the European release-population, extensive spatio-temporal monitoring was carried out. Initially, monitoring was based on sight reports. Since 2014, most individuals have been equipped with GPS-devices, which enabled comprehensive remote monitoring and the implementation of focussed measures against major mortality causes [10,11]. The monitoring was supplemented by extensive data collection on feeding ecology and habitat preference [12,13]. As indicated by a remote sensing study on habitat availability, a suitable habitat for area expansion is plentiful [12]. Since 2011, an increasing number of released Northern Bald Ibises have been migrating independently between the common wintering area in Tuscany and the breeding areas north of the Alps. Since 2012, a steadily increasing number of young birds have hatched in the wild in the breeding areas and followed their conspecifics into the common winter area. This enables extensive observations of the migratory and breeding behaviour of the species [14]. The human-led migration is the main translocation method in this reintroduction project. Chicks from zoos are raised by human foster parents and trained to follow an ultralight aircraft with which they are guided to the wintering area in autumn, where the release takes place. In this way, new migration traditions can be established [9]. The human-led migration provides outstanding opportunities to investigate the flight techniques of this migratory species [11,15,16]. Long-term data on the breeding biology and demography of this release population was analysed and used as a basis for a population viability analysis simulating the possible future development of the population [17]. The simulations indicated that the population needs a minimum size of about 320 individuals for self-sustainability [17]. A comprehensive genetic study as part of the LIFE+ project included an analysis of the remaining wild population as well as zoo and release populations [18]. It forms the framework to establish the available genetic variability in the release population.

### 2.2. Egyptology

Regarding the historical context, analytical and interpretative studies on the Egyptian attitude towards the Northern Bald Ibis and a thorough analysis of the bird’s presence in Egypt have been undertaken for more than 10 years within broader research projects of the Czech Institute of Egyptology, Charles University, Prague. Methodologically, this research covered archaeological excavation, analysis and interpretation of available material. This material encompassed textual and iconographical evidence of the species, textual sources dealing with the ancient Egyptian religious concept of the Akh (represented by a hieroglyphic sign in the shape of the Northern Bald Ibis), collecting (and interpreting) evidence on bodily remains of this avian species from Egypt and, most importantly, building a palaeographic (sign development) database of the Akh (Northern Bald Ibis) depictions from all historical periods of ancient Egypt. All material, pictorial and textual evidence on the Northern Bald Ibis attested from ancient Egypt (from ca. 4000 BCE to ca. 630 CE) was analysed and considered. The textual evidence on the hieroglyphic sign of Akh (written with a picture of the Northern Bald Ibis) was examined and analysed according to specific time periods (e.g., Old Kingdom, Middle Kingdom, New Kingdom etc.). For the development/degradation of the sign, a special palaeographical table was created and used [4]. This enabled us to gather different kinds of data (material, pictorial and textual) on the presence and absence of the Northern Bald Ibis in ancient Egypt.

The present article thus combines primary research (including direct observation and experimentation) on a specific avian species with secondary research into the development and changes of human approaches to the particular species in history. Both research approaches are presented together in each section of the paper. This yields a “synoptic” study covering both types of research.

### 2.3. Ethical Standards

Bird care, keeping, training and release in the frame of the European project followed well established standards in accordance with the legal framework and under the supervision of Waldrappteam Conservation and Research Experts. The weight of the data-logger was well below the recommended maximum value of 5% of the birds’ body weight of about 1.300 Gram. The loggers were fixed on the lower back of the birds via a Teflon-tubes leg-loop harness. This position is known to causes the least drag and minimize also other disadvantages and risks for the carrier (Cyt.). All translocation and management measures have been implemented in the frame of the European LIFE project LIFE+12-BIO_AT_000143. National approvals were provided by the County of Salzburg (21302-02/239/352-2012), Carintia (11-JAG-s/75-2004), Baden-Württemberg (I1-7.3.3_Waldrapp), Bavaria (55.1-8646.NAT_03-10-1) and Italy (0027720-09/04/2013).

## 3. Results

### 3.1. The Appearance

**Figure 1:**
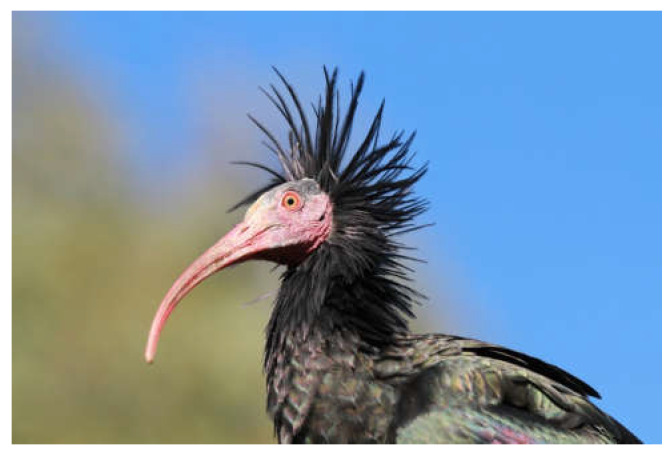
Portrait of an adult Northern Bald Ibis. Photo J. Fritz.

The Northern Bald Ibis is a bird with an exotic appearance featuring a long, curved bill, a naked reddish face, framed by an imposing crest of black lancet feathers, and an all-black plumage that gleams metallic in the sunlight (Figure 1). The Latin name reflects the characteristic appearance: *Geronticus eremita*, elderly hermit. Systematically, the species belongs to the order Pelecaniformes and the family Threskiornithidae. The genus *Geronticus* includes only one more species, the Southern Bald Ibis (*G. calvus*) native to South Africa. The sexes do not differ significantly. The males are slightly heavier than the females (average weight: 1390 g—males, 1257 g—females) [19], with a longer and stronger beak. The distributions of the characteristics overlap, however, and a reliable determination of the sexes is only possible using genetic methods. The juveniles, in contrast, are clearly distinguishable by their grey-feathered head.

### 3.2. The Present State and History

Once a widespread colonial migratory bird species, the Northern Bald Ibis (*Geronticus eremita*; Figure 1) had to be put on the Red List in 1994 as one of the rarest birds in the world. At that time, only one population remained in the wild, living on the Atlantic coast in Morocco. However, even this population was at a critical population size with only 65 breeding pairs [20]. They were the remainders of a species that had large breeding colonies on the African continent from Morocco to Egypt, in large parts of Europe and on the Arabian Peninsula [19].

In Europe, the species went extinct already during the 16th and 17th centuries. The publications by the Swiss naturalist Conrad Gesner [21] and other sources make it possible to reconstruct historical breeding sites located mainly along the northern foothills of the Alps [22,23,24], but there is increasing evidence for a larger former breeding range in Europe, with indications for southern Spain [25], the Upper Adriatic Region [26], Bulgaria [27] or the Kaiserstuhl region in Baden-Württemberg with bones dating to the 4th century AD [28]. Northern Bald Ibis bones found in a cave located in the French department of Ardèche even dated to the Iron Age, between 764 and 406 BCE [29]. The historical evidence for a long-lasting presence of the Northern Bald Ibis in Europe throughout the Holocene corresponds with recent genetic findings. In an extensive analysis of mitochondrial DNA, Wirtz et al. [18] found no genetic differentiation between the Moroccan population and the former Middle East population. This indicates a continuous Northern Bald Ibis population whose breeding range covered large parts of North Africa, Europe and the Middle East.

The separation into a western and eastern population took place when the species disappeared from Europe in the early 17th century. The historical record clearly indicated anthropogenic causes, mainly hunting and collection of chicks [30]. Nonetheless, the rapid decline in Europe was most likely accelerated by the so-called Little Ice Age, as is also evident for other species [31]. The period from the beginning of the worldwide glacial expansion in 1550 until the first climatic minimum in 1650 fits very well with the decline of the Northern Bald Ibis population. The deteriorating climatic conditions may have led to reduced breeding success and higher mortality rate due to the onset of colder climate [26,32]. After vanishing from Europe, the cultural memory of the species was lost for centuries, and historical depictions were taken for portraits of a mythical or symbolic creature. It was not until 1897 that ornithologists recognized that the depictions resemble a real living species that was described for the Middle East, and only then did the species receive its current scientific name, *Geronticus eremita* [23].

At the time the species disappeared from Europe, it was still widespread in the Middle East, with some colonies holding several thousand individuals [33]. By the late 20th century, however, all these colonies had become exterminated. Major causes included the destruction of its habitat, the disturbance of the breeding colonies and the industrialization of agriculture. A well-documented example is a former colony along the Euphrates River, near the town of Bireçik in southern Turkey. There, the intensive application of DDT against malaria and locusts caused the loss of more than 600 individuals, about 70% of the population, between 1959 and 1960 [34,35]. In 1989, of the three remaining adult birds that returned from migration to this breeding site, only one survived to the end of the breeding season. This was generally assumed to be the end of the last wild colony in the Middle East [36], but in fact it was not. Very unexpectedly, a small relict population, comprised of only seven individuals, was discovered in 2002 near Palmyra in Syria [37]. Satellite tracking revealed that they still migrated over more than 3000 km to the historical wintering site near Addis Ababa in Ethiopia [38]. The same birds, which behaved very shyly at their breeding site in the Syrian Desert, lived in an agro-pastoral landscape in the close surroundings of villages in Ethiopia during winter [39].

Extensive international conservation efforts followed the surprising discovery of this relic population. They even included the release of three juveniles from a semi-captive breeding colony in Bireçik, Turkey, in 2010 [40]. Ultimately, however, all efforts were in vain. The last bird disappeared in 2013 and with it also the last historical migration tradition [41]. This event also marks the general extinction of the Northern Bald Ibis in its characteristic lifestyle as a migratory species. There is no longer any evidence that a migrating population still exists anywhere in the former distribution area.

What remains after the vanishing of this extraordinary bird are cultural traces that can be found throughout the historical area. The richest and most exciting traces are from ancient Egypt. There, the Northern Bald Ibis was inseparably liked with the concept of the Akh (often translated as “the blessed dead” or “effective spirit”) [42,43,44,45,46,47,48]. The hieroglyphic sign of the word Akh was written as a picture of this bird. As the word *Akh* was very important for the ancient Egyptians, the sign in the shape of the Northern Bald Ibis is abundant in Egyptian texts and iconographic sources. The bird and the *Akh*-spirit (in the shape of the bird) were linked to the same habitat by ancient Egyptians. Direct observation showed that the birds formerly dwelled on limestone cliffs on the eastern side of the Nile valley. This very region, called Akhet (i.e., the horizon) by the Egyptians, was also believed to be the dwelling place of the blessed spirits and gods [42,49,50,51,52] (see the Appendix A).

### 3.3. Historical Images and Cultural Significance

**Figure 2:**
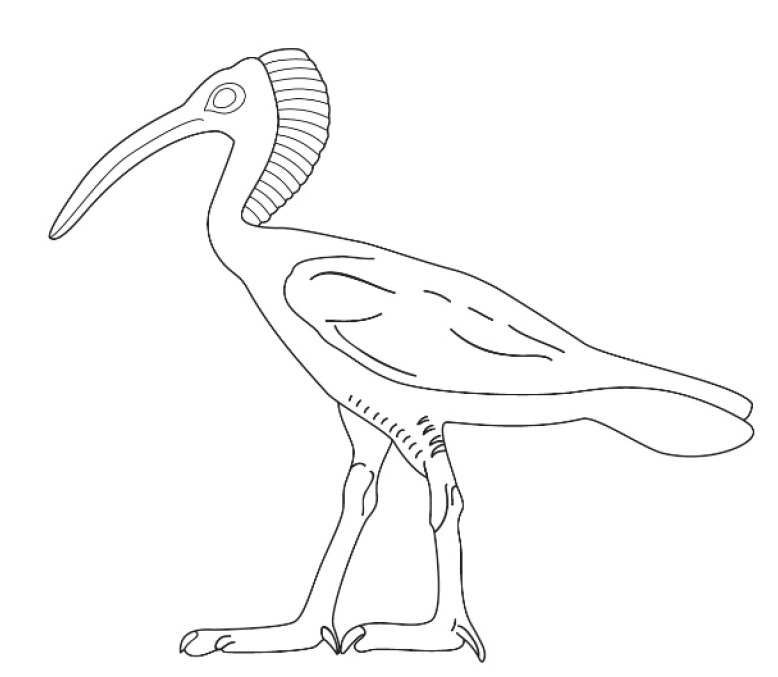
The hieroglyphic Akh-sign from the tomb of Akhethotep (ca. 2400 BCE). Drawing by Lucie Vařeková.

The earliest evidence for the cultural meaning of the Northern Bald Ibis stems from ancient Egypt, where the species was used as a pictorial representation for the hieroglyphic sign Akh (Figure 2). The sign, like its living model, is easily recognizable by the long curved bill and a typical crest covering the back of the head [4]. The most surprising fact about the Northern Bald Ibis in ancient Egyptian sources is the almost complete lack of material evidence that does not match the abundance of pictorial representations. It is precisely this inconsistency of evidence that attracted the attention of scholars and started their research on the presence of the bird in ancient Egypt and its early disappearance from the country after a climate change followed by high human activity in the bird’s foraging areas (see the Appendix B).

Most historical drawings of the Northern Bald Ibis represent adult birds with a bare head and the feather crest [7,19,23,48]. A noticeable exception is a drawing by Conrad Gessner [21] showing a juvenile bird with a small crest and a completely feathered head. Even more clear is a 15th-century altarpiece from around Munich, Germany, which represents the Mount of Olives scene with Christ and the disciples. A juvenile Northern Bald Ibis is depicted at the edge of this picture, with a worm in its bill as characteristic food. Such elaborate representations indicate that the artists knew the birds first-hand, an important indication for the presence of the species in these times. The bird in the altar scene can be interpreted as a representation of suffering, death or the hereafter [5,23]. Christian cultures in Europe regarded black birds in general as a rather bad omen or nuisance [26]. Nonetheless, this rather negative mythological image most likely did less harm to the species than its reputation as a tasty food bird. Gesner [21] reported that the Northern Bald Ibis was praised as food and considered a treat because of its lovely flesh and soft bones. They were caught or shot, and the pre-fledged juveniles were taken from the nest [30].

The bird’s importance for the dining tables of the nobility and the clergy even led to protective measures as the populations declined. In Salzburg (Austria), for example, the archbishop’s decrees of 1504 and 1584 criminalized the shooting of Northern Bald Ibises from the Mönchsberg cliff above the city of Salzburg. In the same century, Emperor Maximilian I provided artificial nesting aids in the rock walls in Graz. In the same period, an order was issued in the city of Graz, Austria, where a colony also occurred, that Northern Bald Ibises should not be shot but rather cherished and guarded [33,53]. Even these measures did not prevent extinction. The last evidence for Bald Ibis occurrence in Europe comes from Graz in 1621 [30].

In the Arab region, the Northern Bald Ibis enjoyed a more positive and more significant cultural image than in the Christian world. In the Muslim tradition, the birds were considered semi-sacred for two main reasons. According to the tradition, it was a Northern Bald Ibis that led the Quranic patriarch Nuh (the Biblical Noah) and his family to the fertile lowlands of the Euphrates River after a successful landing of the Ark on Mount Ararat [8]. In the Turkish town of Bireçik (where a Northern Bald Ibis colony nested until the late 1990s), local people honoured these birds as symbolic pilgrims to Mecca and leaders of the *hajj*. Once a year, the Northern Bald Ibises of Bireçik flew southwards in large numbers, just like pilgrims to Mecca, and they returned in spring after a period common also for pilgrimages [5,33]. For that reason, the Bireçik residents used to celebrate the return of the birds in February with the traditional Kelaynak festival (*kelaynak* is the Turkish name for the species). Presumably for these reasons, this ibis species was less pursued as food in the Arab world.

### 3.4. Foranging and Habitat

A remarkable characteristic of the Northern Bald Ibis is its long and fragile curved bill. It is poorly suited to hunt for mobile prey but perfectly shaped to dig deeply for invertebrates in the soil. This bird is therefore mainly a tactile hunter. Its preferred habitat is open landscapes with low vegetation and an abundance of soil fauna. Under favourable conditions, its diet consists predominantly of worms and larvae [13,54], but the birds show high flexibility in their feeding habits. In the Syrian Desert, for example, they feed mainly on tadpoles, which they pick up from the beaches of man-made reservoirs [55], whereas at the wintering site, the same individuals predominantly dig for worms and larvae at freshly cut hayfields [56]. Lizards represent an essential part of the diet of the remaining population in Morocco [57].

The flexibility in foraging is also reflected in a diversity of foraging habitats. Northern Bald Ibises once fed in the Syrian Desert [58], in the Moroccan steppe [57,59], on meadows and pastures of the northern foothills of the Alps [12,13,54] or the Ethiopian highlands [56] and even on Turkish mint fields [60]. A common characteristic of these feeding habitats is that they are rather open landscapes with loose vegetation coverage. The birds clearly prefer low vegetation, usually not higher than 10 cm. This may be a natural characteristic of the vegetation, especially in semi-arid areas, but in most regions the birds benefit from grazing or mowing [13,59]. Moreover, even though the Northern Bald Ibis is generally not a wetland inhabitant like many other ibis species, it needs a freshwater source for drinking and bathing. This became evident in Morocco, where the provision of freshwater near the breeding colony significantly enhanced breeding productivity [61].

A noticeable peculiarity in connection with the feeding ecology is the frequent proximity of the breeding habitats to human settlements. This is evident from historical reports for the former European population [21,22,23,26] but also known for most former breeding sites in the Moroccan and the Algerian Atlas [7,59], in Turkey [34,36] and for the former wintering site in Ethiopia [55]. The assumption is that the presence of the species in various regions was dependent on human beings who cleared or drained the land and kept it open through farming or grazing [22,23,26,59]. This represents a kind of mutualism because the birds benefited from the open sites and ate the larvae of pest insects in return. As with many other species, however, proximity to humans ultimately also carries a high risk for the animals, depending on the respective culture and period.

**Figure 3:**
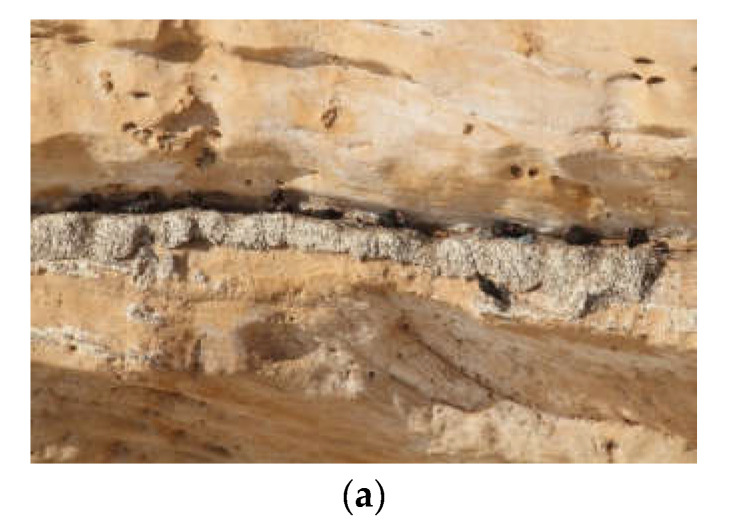

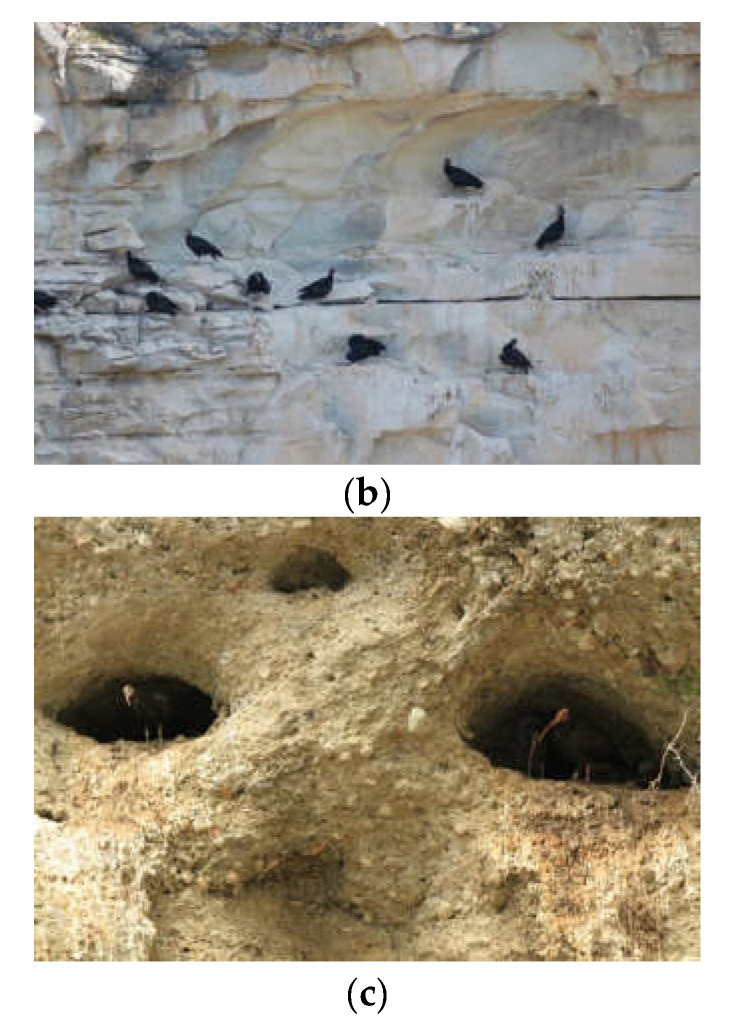
Various Northern Bald Ibis breeding cliffs; (**a**) Agadir, Morocco (photo D. Tome); (**b**) Birecik, Turkey (photo J. Fritz); (**c**) Kuchl, Austria (photo J. Fritz).

The breeding sites were often close to human settlements, too, but this was most likely more a coincidence than a mutualistic relationship. The Northern Bald Ibis as a colonial breeder that needs cliffs that are structured with niches or ledges (Figure 3). Many such cliffs consisted of limestone or conglomerate located on the coast, along large rivers or on lake shores. These were often also preferred areas for establishing human settlements. Examples include the former Turkish colony in Bireçik on the Euphrates River or former European colony sites such as Salzburg on the Salzach River, Passau on the Danube or Ueberlingen at Lake Constance [22]. This coincidence also contributed to the extirpation, indirectly through disturbance and destruction of the breeding sites as well as directly through hunting and collection of nestlings from the nests.

There is almost no material evidence on the habitat use of the Northern Bald Ibis in ancient Egypt. The only available evidence comes from textual and iconographic sources of religious nature. For example, a study on the meaning and cultural context of the Akh sign provided us with information about the bird’s habitat (cliffs of limestone massifs at the eastern bank of the Nile), character (gregarious, migratory bird) and flight characteristics during migration [4]. However, the inseparable link between the Northern Bald Ibis and its habitat (limestone cliffs) lay behind the significance of the bird in ancient Egyptian culture and religion, as the eastern limestone cliffs were believed to be the domain of gods and spirits of the ancestors (see the Appendix A).

### 3.5. Behavioural Characteristics

The Northern Bald Ibis is a year-round social species with hierarchically structured colonies consisting of up to several thousand individuals. The species is monogamous, with some couples forming long-lasting bonds while others remain together only for a breeding season. Both partners breed and raise the chicks, a behaviour characteristic of many monomorphic species [62]. The fledging rate varies considerably among the populations, ranging from 1.0 to 2.2 chicks per nest depending on site and conditions [17]. According to data from the European project, the family groups dissolve already in the breeding area. The juveniles join together in groups that follow experienced conspecifics to the wintering site [10].

The species has a rather moderate repertoire of calls. Most common is the “croop” call, which is used in an affiliative context—the characteristic greeting behaviour with the rhythmic vertical movement of the beak—as well as during agonistic encounters [63]. The “croop” has highly variable temporal and structural parameters, pointing to the expression of affective states and even encoding individual differences that enable individual recognition [64]. This is accompanied by an equally distinguishing morphological characteristic: the conspicuous bare head. In adult birds, the pattern of the dark areas allows individual recognition, even by humans [65]. In males, moreover, the bare throat area varies in size between individuals and apparently has hormonally controlled variation in the intensity of the red colouration [66]. Based on those features, the bare head of the Northern Bald Ibis most likely evolved mainly in the context of social interaction and mate choice. There is also some evidence that, in species like the Northern Bald Ibis inhabiting hot environments, bare skin has evolved to dissipate heat [67].

**Figure 4:**
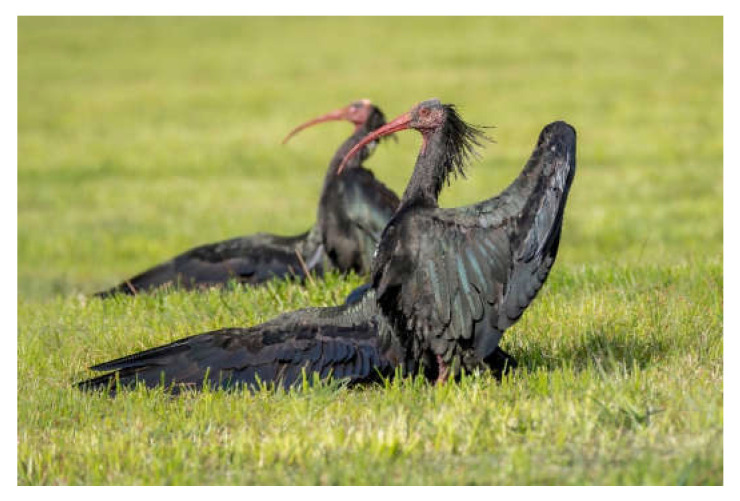
Sunning behaviour. Photo R. Beck.

A noticeable and characteristic behaviour of the Northern Bald Ibis, as of other ibises (Threskiornithidae), is the sunning (or “sun-bathing”) behaviour, in which the bird remains in a stiff upright position with its wings outstretched (Figure 4). Such “sun worship” has contributed to the veneration of this species in various cultures [8], a notion that might have been strengthened by the bird’s ties to the idea of the eastern horizon in ancient Egypt [46,50]. However, the actual function of this behaviour remains unclear [68]. Hypotheses include thermoregulation, killing of ectoparasites by heating the feathers and the body surface, or vitamin D-synthesis by exposing bare skin on the underside of the wing to the sun. In any case, sunning behaviour has a clear social component. When one bird begins, it usually stimulates other conspecifics, which makes this behaviour even more conspicuous.

### 3.6. Migration

The species was migratory over its entire historical range, with known wintering sites along the African west coast down to Mauritania and Mali and along the African east coast down to Ethiopia and Eritrea [4,10,38,57]. The former European population is known to have left in autumn and return in spring, but no evidence for the migration pathway or the historical wintering site is available [22,23]. The migratory European release population migrates to a wintering site in southern Tuscany. This release site, however, was selected based both on historical records and on the current availability of suitable habitats [10,12]. Under appropriate ecological and climatic conditions, colonies of various migratory species are known to shorten their migration route or change to a resident lifestyle. This occurs especially along coastlines with year-round moderate climatic conditions [69]. This is also valid for the Northern Bald Ibis. Several resident colonies are known along the Atlantic coast in Morocco, but such colonies most likely also existed along the Red Sea and in ancient times also along the Nile (see Appendix A). In the meantime, all migrating colonies have been eradicated, while two residential colonies on the Atlantic coast still exist [57,59].

Due to the extinction of all migratory colonies, most of our knowledge about the species-specific migration behaviour comes from the migratory European release population. These birds are descendants of former migratory colonies in the Moroccan Atlas. Research on their physiology, energetics and behaviour has shown that this bird is an enduring migratory species with a pronounced navigation ability. In Europe, they enter into a migratory state (*Zugbereitschaft*) in early August [70,71]. At that time, they leave their breeding areas and usually return in the next season beginning in late March [72]. The Middle East colonies (mainly Bireçik and Palmyra) had their rhythm shifted by about one month, with a departure in early July and return from late February on [19,37].

### 3.7. Endangerment and Disappearance

In 2018, the down-listing of the species on the IUCN Red List from critically endangered to endangered was justified by major conservation action to secure the breeding sites in Morocco. However, this decision was controversial. Although the population has developed well in recent years and its breeding area shows signs of expansion [73], it remains the world’s only wild population so far, spatially restricted to a small area on the Atlantic coast in Morocco. The significant decline of the population in 1996 as a result of an epidemic [74] and its dependence on management measures such as the provision of supplementary freshwater [61] indicate that this single population cannot ensure the permanent survival of the species.

Moreover, it must be assumed that this population faces an increasing threat due to the ongoing climate change. Morocco is among the countries that are expected to face the strongest effects in terms of the rise in temperature, decrease in precipitation and weather extremes, and the coastal region will most likely be particularly affected [75]. This will put increasing pressure on the Moroccan population. Under these changing conditions, the residential lifestyle of this coastal population may become detrimental because residential populations lack ecological flexibility related to migration behaviour. There is also hardly any evidence that secondary residential populations are able to change back to a migratory lifestyle [69,76,77].

Due to the lack of physical evidence on the presence of the Northern Bald Ibis in Egypt, one can only use pictorial and textual evidence in researching the nature of the bird’s presence in the country. In this respect, the pictorial evidence on the species was much more accurate, precise and elaborate in the early periods of Egyptian history, until the final phase of the third millennium BCE [4]. In later times, the representations become very schematized, sometimes only minimally corresponding to their natural model. Moreover, there is no material, pictorial or textual evidence for keeping, breeding, hunting, killing, sacrificing and, above all, for the mummification of the Northern Bald Ibis in Egypt in any period [78,79]. This stands in striking contrast to the Sacred Ibis (*Threskiornis aethiopicus*) and the Glossy Ibis (*Plegadis falcinellus*), which are known to have been kept and mummified in huge numbers [78,80]. Judging from this iconographic evidence and the complete absence of material evidence (skeletal remains and mummies) in the final phase of the third millennium BCE, the Northern Bald Ibis had presumably started to disappear from Egypt or alter its migration routes at or slightly before that time. Disappearance from ancient Egypt seemingly followed a period of swift desiccation of the land and expansion of arid areas that occurred in the first half of the third millennium BCE, when other species such as the elephant, the giraffe or the Saddle-Billed Stork are also known to have disappeared [81,82,83,84]. As opposed to the other species, which disappeared during the time of climate change and gradual desiccation, the Northern Bald Ibis apparently disappeared 500 years later.

## 4. Discussion

The Northern Bald Ibis is an iconic species for both ornithology and Egyptology. The interdisciplinary approach enables tracing the bird’s presence, habits and habitats almost 5000 years back in history. Coincidently, this ibis species is connected with the notion of death and disappearance in both scientific fields. For ornithologists and conservationists, it is best known for being one of the most endangered avian species in the world and a model for current conservation and reintroduction attempts [9,10,19]. In ancient Egypt, it was linked with the concept of the blessed dead (called the Akh), who disappeared from the world of the living but returned back to (after)life after a successful journey through the underworld [48].

Recent studies on this bird’s behaviour and ecology, particularly in the context of the European LIFE project, enriched Egyptology with information about the species’ shape, colouring, habitat, social habits, migration periods, routes and formations. With these data, Egyptologists were able to clarify their views on key cultural and religious concepts [4,48,85].

In turn, in-depth Egyptological studies proved to be valuable for recent research and conservation attempts. Tracing the history of the Northern Bald Ibis several millennia into the past yields significant information about the coexistence of this species and the human population in very different epochs and regions. Such coexistence and relationship worked well for long periods of time, but at some point the situation changed to the disadvantage of the birds. This was the case in ancient Egypt during the final phase of the third millennium BCE [4], in Europe during the Middle Ages [22,23], and in most other parts of their former breeding range during the last century [19,38,59].

In fact, the cultural and historical traces of the disappearance of the Northern Bald Ibis from ancient Egypt are similar to those of the Saddle-Billed Stork. These can thus be used for direct comparison. The Egyptians used the image of the Saddle-Billed Stork as a hieroglyphic sign *ba* (meaning “majestic”, “powerful” or even “divine”). When the species disappeared from Egypt after a climate change early in the third millennium BC, the hieroglyphic *ba* sign first lost its accuracy and slowly ceased to resemble the stork at all. Then the sign and the Egyptian term *ba* was given a new meaning (“soul”) and, finally, a completely new hieroglyphic sign (a human-headed falcon) was invented for the *ba* [4,85]. Thus, the climate change of the early third millennium BCE had a direct impact not only on nature, human life and society but also on such Egyptian culture and religion.

Further Egyptological research revealed that the Northern Bald Ibis disappeared from Egypt during the late third millennium BC, some 500 years after a major climate change had occurred in the region [4]. Ongoing research indicates that the most important factor for the disappearance was changing human activity in and around the bird’s feeding and breeding areas because of the ongoing climate change. This included the need for new irrigation projects, increased quarrying and building activity, as well as social unrest and struggles stemming from social disorder after the collapse of the Egyptian state at the end of the Old Kingdom (the second half of the third millennium BCE).

Disappearance of the Northern Bald Ibis in the final phase of the third millennium BCE, in the 16th century AD and in the 20th and 21st centuries occurred in different regions and were caused by a different trigger event. However, they have one important factor in common: Intensified human activity directly or indirectly forcing this bird species to alter its habits and habitat, to change or leave its breeding areas or migration routes. The extinction of the ancient Egyptian population and the European population also correlates with marked changes in the climate [86]. In the present day, climate change is also expected to pose growing threats to the last remaining wild population in Morocco.

Noteworthy in this context is the International Single Species Action Plan for the Conservation of the Northern Bald Ibis, which was published in 2015 and foreseen for a 10-year period [87]: the term climate change does not appear once. This is a deficiency given the historical coincidence between population extinctions and changes in climate presented in this paper. It also ignores the fact that the ecosystems in the Middle East and northern Africa, which the Action Plan outlines as prioritized areas for the conservation and translocation measures, are assumed to be disproportionally affected by climate change effects [88].

In Europe, which lies geographically outside the prioritized areas of the action plan, two successful reintroduction projects are being implemented. *Proyecto Eremita* in Andalusia, Spain, is on the way to establishing a residential colony [19], while Waldrappteam is establishing a migratory colony with breeding colonies on the northern and southern Alpine foothills and a common wintering site in southern Tuscany [5,10]. This is currently the only migratory population of this species. Recent population viability analysis indicates that the migratory release population is relatively stable against the stochastic events that are expected to occur with increasing frequency due to climate change [17]. An analysis of foraging areas in the northern Alpine foothills also indicates a rich and sustained availability of suitable foraging habitats [12].

Based on the population and habitat modelling, from 2022 to 2028 a second LIFE project is implemented (LIFE20 NAT/AT/000049). This project, covering four countries (GER, AUT, CH, IT), aims for a self-sustaining population with at least 360 individuals, divided into 7 breeding colonies at the northern and southern foothills of the Alps and a common wintering site in southern Tuscany. Extensive campaigns are aimed at substantially and sustainably reducing losses from the primary causes of death, which are illegal hunting in Italy and electrocution in the breeding areas. Based on the results of the interdisciplinary investigations and the modelling, climate change is seen as the greatest risk to sustainable reintroduction and various mitigation and compensation measures are envisaged accordingly. This includes, among others, the subdivision of the population into several colonies with breeding areas in different climatic regions, optimization of genetic variability and intensive monitoring. However, the extensive experience with this European release population in combination with the historical facts as presented in this paper should also help to initiate and optimize conservation measures in the entire historical distribution area, especially with regard to the apparent sensitivity of the species to climate change.

This interdisciplinary study illustrates the importance of tracing the cultural-historical meaning of a species and human–animal interactions with a special focus on epochs of instability and change. The importance of historical evidence should be understood less in a purely geographical context, as is the case in the current action plan for the Northern Bald Ibis, but rather be included in the assessment of the ecological, cultural and socio-economic framework for species protection measures. The analysis also points to the clear sensitivity of the species to climatic changes and the resulting ecological and cultural framework conditions. Therefore, the assessment, planning and implementation of species protection measures must not be based on the assumption of static ecological and cultural framework conditions. Rather, a dynamic approach is required, such as through suitable modelling [17]. This is a logical general requirement when working in and with biological systems. Importantly, the current climate change makes flexible approaches a prerequisite for the sustainable success of species conservation measures. Finally, our interdisciplinary study shows that knowledge from current scientific research and experience in ongoing species protection measures can promote and inspire cultural-historical research in a significant way. The authors’ shared hope is that the successful return of this charismatic bird species will continue to enrich human culture in the future. Only then would this iconic bird and the ancient Egyptian Akh once again share the same meaning—to be gloriously resurrected from the dead.

## Figures and Tables

**Figure A1:**
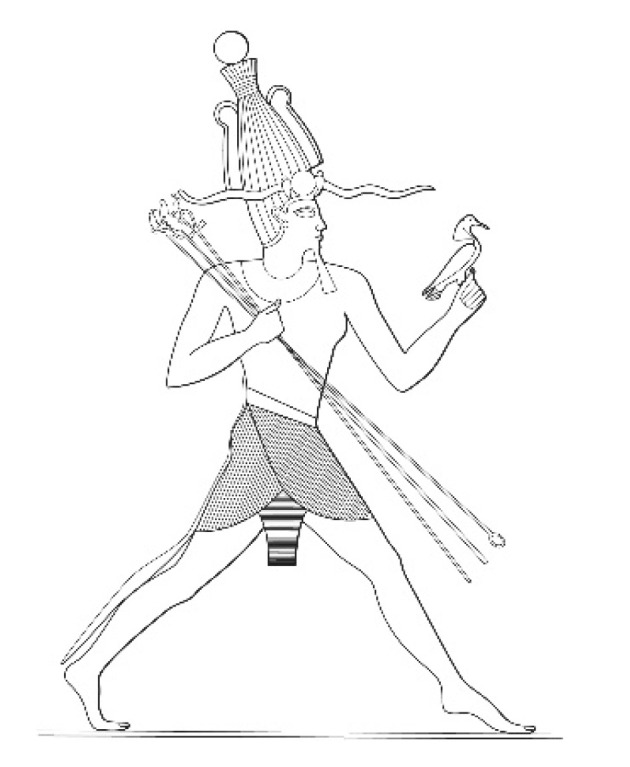
Egyptian king during the so-called *Vogellauf* ritual. Drawing by Lucie Vařeková.

## Data Availability

The data presented in this study are available on request from the corresponding author. Spatio-temporal data of the European Northern Bald Ibis release population are publicly available at Movebank (https://www.movebank.org/cms/movebank-main).

## Appendix A

### The Northern Bald Ibis in Ancient Egyptian Culture and Religion

In ancient Egypt, the image of the Northern Bald Ibis was inseparably linked with the notion of Akh, often translated as the “blessed dead” or “effective spirit” [4,42,43,44,45,46,47]. The hieroglyphic sign of the word Akh was written as a picture of this bird. The basic meaning of the term Akh related to effectiveness and a reciprocal relationship that crossed the borderlines between different cosmic spheres, between the world of the living and the world of divine beings [47]. The ancient Egyptians considered their blessed, efficient and influential dead (the Akhu, i.e., the plural for of Akh) “transfigured”, but a deceased human had to first be admitted and elevated to this new state. When a dead person’s journey to the afterlife was successfully completed and he/she was justified and transfigured into an Akh, that person became a mighty and mysterious entity that participated in the divine sphere of existence and yet still had some influence upon the world of the living [48,50,51,89,90]. A very important role in the process of becoming an Akh was reserved for the horizon (Akhet). Although it mainly represented the junction of cosmic realms (the earth, the sky and the netherworld), the horizon was a region in itself [49], and it was believed to be the place of the sunrise and resurrection, a region where divine beings dwelled and where they interacted with the world of the living [44,50,51,52,89,90,91].

In Egypt, the horizon (the Akhet) was represented by rocky cliffs at both sides of the Nile Valley. The Northern Bald Ibis nested precisely on these limestone cliffs on the eastern bank of the Nile, which (as the “eastern horizon”) was considered the realm of gods and ancestral spirits by the Egyptians. The habitat of the Northern Bald Ibis thus represented the main reason for the Egyptians to believe that these birds represent the souls of their blessed ancestors. After a successful transfiguration into an Akh, the deceased still needed to interact with the living, since it was the latter who performed rituals, carried out the embalming and funerary requirements and provided their dead ancestors with offerings [36,40]. The Akhu, on the other hand, provided the living with divine protection and supernatural help, according to the faith of ancient Egyptians. Thus, the Akh-society and the living represented co-dependent communities, and their mutual relationships and cooperation formed one of the pillars of ancient Egyptian religion [38,42].

## Appendix B

### The Presence of the Northern Bald Ibis in Ancient Egypt

Dealing both with the presence and the disappearance of the species in ancient Egypt, the Egyptological research paradoxically benefits from the almost complete absence of primary material evidence (bones, other bodily remains or mummies) on the species in ancient Egypt, which does not match with abundant pictorial evidence.

Remarkably, there are no attestations of keeping, hunting, sacrificing or mummifying the Northern Bald Ibis, contrary to the Sacred Ibis (*Threskiornis aethiopicus*) or the Glossy Ibis (*Plegadis falcinellus*). The absence of material evidence combined with a gradual degradation of secondary evidence (script, iconography) serves as proof for the early disappearance of the species. The only attested piece of material evidence for the Northern Bald Ibis in Egypt in the form of skeletal remains comes from the Maadi region [79] located south of modern Cairo where the so-called Maadi culture had its settlements around 4000–3400 BCE. This unique find represents both the earliest evidence for the presence of the bird in Egypt and (very surprisingly) its only confirmed preserved bodily remains. Pictorial representations of the bird have been recorded only from later periods of Egyptian history. The earliest Egyptian depiction is attested on the so-called Ibis slate palette dated to the Naqada III Period (circa 3300–3000 BCE), other early examples date to the Late Predynastic and Early Dynastic Periods (c. 3000–2700 BCE). From the Old Kingdom (c. 2700–2180 BCE) onwards, a pictorial representation of the Northern Bald Ibis was used as a hieroglyphic sign for the word Akh, with only a few possible exceptions [92,93]. Some Old Kingdom depictions of the bird show a very high accuracy, revealing the precise observation skills of the ancient scribes and artists. This proves that the ancient scribes or artists in Egypt were very well aware of the typical features of the bird. In contrast, Egyptian representations of the Northern Bald Ibis in later periods, e.g., from tombs dating to the Middle Kingdom (c. 2130–1770 BCE) are rather schematic and less accurate and detailed than earlier examples [4,94].

In the time of the New Kingdom (c. 1550–1070 BCE), as well as in the following periods of Egyptian history (until the first centuries CE), Egyptian pictorial representations of the Northern Bald Ibis are limited to the hieroglyphic Akh-sign (in the form of the bird’s rather stylized depiction) and to the depiction of a mysterious ritual called the *Vogellauf* in Egyptology [85,95]. The representations of the ritual show the king running with a Northern Bald Ibis in one hand and three symbolic sceptres of prosperity in the other (Figure A1). Based on ornithological evidence and comparative data proving an early disappearance of this ibis species from Egypt, Egyptologists were able to ascertain that the *Vogellauf* images were merely symbolic, as the bird “held” by the king was already extinct at the time when the scene was carved into the walls of Egyptian temples [85].

Moreover, ancient Egyptian texts (mostly of cultic nature) bear also some useful information of the Northern Bald Ibis migration. In the royal tombs of the New Kingdom, migratory birds coming from the north are viewed as messengers from the hereafter, i.e., souls of the ancestors. The society of the blessed dead (Akhu) also had its own hierarchy structured similarly to the world of the living, the deceased king being at the top of the Akh spirits. The strictly hierarchical structure of the Akh society with only one entity at the very top may have not only reflected the structure of human society but also stemmed from the well-observed V-formation used by the birds during migration. It is noteworthy, that the study of the ancient Egyptian population and cultural significance of the Northern Bald Ibis is particularly important because very different reasons must have been behind the bird’s disappearance from ancient Egypt. None of the above-mentioned probable causes for the species extinction in 16th century Europe (Little Ice Age or hunting) and in the modern Middle East (DDT) had occurred in Egypt in the 2nd millennium BCE.
